# Cluster of Coronavirus Disease Associated with Fitness Dance Classes, South Korea

**DOI:** 10.3201/eid2608.200633

**Published:** 2020-08

**Authors:** Sukbin Jang, Si Hyun Han, Ji-Young Rhee

**Affiliations:** Dankook University Hospital, Dankook University College of Medicine, Cheonan, South Korea

**Keywords:** respiratory infections, severe acute respiratory syndrome coronavirus 2, SARS-CoV-2, SARS, COVID-19, 2019 novel coronavirus disease, coronavirus disease, zoonoses, viruses, coronavirus, South Korea

## Abstract

During 24 days in Cheonan, South Korea, 112 persons were infected with severe acute respiratory syndrome coronavirus 2 associated with fitness dance classes at 12 sports facilities. Intense physical exercise in densely populated sports facilities could increase risk for infection. Vigorous exercise in confined spaces should be minimized during outbreaks.

By April 30, 2020, South Korea had reported 10,765 cases of coronavirus disease (COVID-19) (*1*); ≈76.2% of cases were from Daegu and North Gyeongsang provinces. On February 25, a COVID-19 case was detected in Cheonan, a city ≈200 km from Daegu. In response, public health and government officials from Cheonan and South Chungcheong Province activated the emergency response system. We began active surveillance and focused on identifying possible COVID-19 cases and contacts. We interviewed consecutive confirmed cases and found all had participated in a fitness dance class. We traced contacts back to a nationwide fitness dance instructor workshop that was held on February 15 in Cheonan.

 Fitness dance classes set to Latin rhythms have gained popularity in South Korea because of the high aerobic intensity (*2*). At the February 15 workshop, instructors trained intensely for 4 hours. Among 27 instructors who participated in the workshop, 8 had positive real-time reverse transcription PCR (RT-PCR) results for severe acute respiratory syndrome coronavirus 2, which causes COVID-19; 6 were from Cheonan and 1 was from Daegu, which had the most reported COVID-19 cases in South Korea. All were asymptomatic on the day of the workshop. 

By March 9, we identified 112 COVID-19 cases associated with fitness dance classes in 12 different sports facilities in Cheonan (Figure). All cases were confirmed by RT-PCR; 82 (73.2%) were symptomatic and 30 (26.8%) were asymptomatic at the time of laboratory confirmation. Instructors with very mild symptoms, such as coughs, taught classes for ≈1 week after attending the workshop (Appendix). The instructors and students met only during classes, which lasted for 50 minutes 2 times per week, and did not have contact outside of class. On average, students developed symptoms 3.5 days after participating in a fitness dance class (*3*). Most (50.9%) cases were the result of transmission from instructors to fitness class participants; 38 cases (33.9%) were in-family transmission from instructors and students; and 17 cases (15.2%) were from transmission during meetings with coworkers or acquaintances. 

**Figure:**
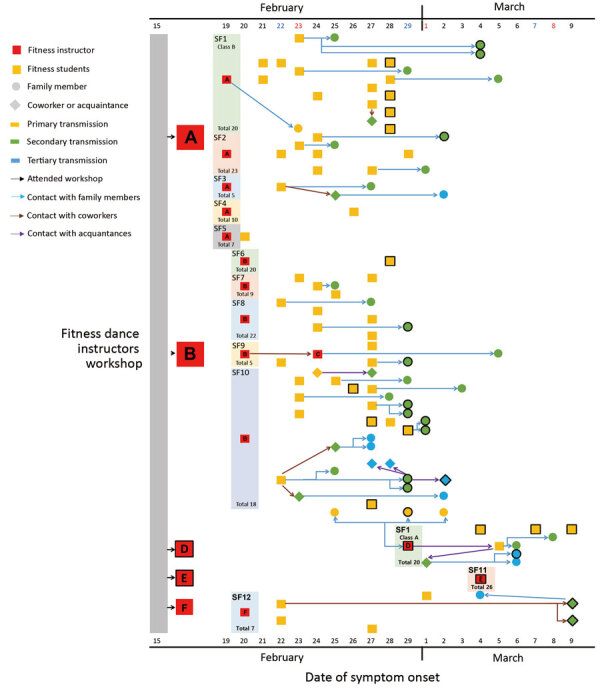
Case map of confirmed coronavirus disease (COVID-19) cases associated with fitness dance classes in Cheonan, South Korea, by date of symptom onset and relationship. Instructors outside of Cheonan are excluded. In 7 cases, transmission was suspected in the presymptomatic phase and the longest period before symptom onset was 5 days. None of the instructors had COVID-19 symptoms on the day of the workshop, but instructors from Daegu, which recently had a large outbreak, developed symptoms 3 days after the workshop. Sports facilities are represented by bars on the left with the number of students per class included. Bold outlines indicate a positive test for COVID-19 in a person in the presymptomatic phase.

Among 54 fitness class students with confirmed COVID-19, the median age was 42, all were women, and 10 (18.5%) had preexisting medical conditions (Appendix Table 1). The most common symptom at the time of admission for isolation was cough in 44.4% (24/54) of cases; 17 (31.5%) case-patients had pneumonia. The median time to discharge or end of isolation was 27.6 (range 13–66) days after symptom onset.

Before sports facilities were closed, a total of 217 students were exposed in 12 facilities, an attack rate of 26.3% (95% CI 20.9%–32.5%) (Appendix Table 2). Including family and coworkers, transmissions from the instructors accounted for 63 cases (Appendix Figure 2). We followed up on 830 close contacts of fitness instructors and students and identified 34 cases of COVID-19, translating to a secondary attack rate of 4.10% (95% CI 2.95%–5.67%). We identified 418 close contacts of 34 tertiary transmissions before the quarantine and confirmed 10 quaternary cases from the tertiary cases, translating to a tertiary attack rate of 2.39% (95% CI 1.30%–4.35%).

The instructor from Daegu who attended the February 15 workshop had symptoms develop on February 18 and might have been presymptomatic during the workshop. Evidence of transmission from presymptomatic persons has been shown in epidemiologic investigations of COVID-19 (*4*,*5*).

Characteristics that might have led to transmission from the instructors in Cheonan include large class sizes, small spaces, and intensity of the workouts. The moist, warm atmosphere in a sports facility coupled with turbulent air flow generated by intense physical exercise can cause more dense transmission of isolated droplets (*6*,*7*). Classes from which secondary COVID-19 cases were identified included 5–22 students in a room ≈60 m^2^ during 50 minutes of intense exercise. We did not identify cases among classes with <5 participants in the same space. Of note, instructor C taught Pilates and yoga for classes of 7–8 students in the same facility at the same time as instructor B (Figure; Appendix Table 2), but none of her students tested positive for the virus. We hypothesize that the lower intensity of Pilates and yoga did not cause the same transmission effects as those of the more intense fitness dance classes. 

A limitation of our study is the unavailability of a complete roster of visitors to the sports facilities, which might have meant we missed infections among students during surveillance and investigation efforts. Discovery of outbreak cases centered on exercise facilities led to a survey of instructors who participated in a fitness dance workshop and provided clues to identifying additional cases among students. Early identification of asymptomatic persons with RT-PCR–confirmed infections helped block further transmissions. Because of the increased possibility of infection through droplets, vigorous exercise in closely confined spaces should be avoided (*8*) during the current outbreak, as should public gatherings, even in small groups (*9*,*10*).

Appendix Additional information about a cluster of coronavirus disease associated with fitness dance classes, South Korea.
