# Supplementary material for: Cluster of Coronavirus Disease Associated with Fitness Dance Classes, South Korea
Source: Emerg Infect Dis. 2020 Aug;26(8):1917–20. doi: 10.3201/eid2608.200633 (PMC7392463; doi:10.3201/eid2608.200633)
Supplement: Appendix — Additional information about a cluster of coronavirus disease associated with fitness dance classes, South Korea. [file 20-0633-Techapp-s1.pdf]

Article DOI: https://doi.org/10.3201/eid2608.200633

# Cluster of Coronavirus Disease Associated with Fitness Dance Classes, South Korea

## Appendix

**Appendix Table 1.** Clinical characteristics of 54 persons with diagnosed coronavirus disease who participated in fitness dance classes, South Korea*

| Characteristics | No. (%) |
| --- | --- |
| Age, y (mean ± standard deviation) | 41.6 ± 10.2 |
| Sex | |
| M | 0 |
| F | 54 (100) |
| Preexisting conditions | 10 (18.5) |
| Hypertension | 4 (7.4) |
| Diabetes | 4 (7.4) |
| Chronic obstructive pulmonary disease | 1 (1.9) |
| Congestive heart failure | 0 |
| Chronic kidney disease | 1 (1.9) |
| Chronic liver disease | 2 (3.7) |
| Malignancy | 2 (3.7) |
| Body mass index, kg/m$^2$† | 22.8 ± 3.04 |
| Symptoms at admission | |
| Fever (>37.5°C) | 13 (24.1) |
| Chills | 17 (31.5) |
| Cough | 24 (44.4) |
| Sputum | 22 (40.7) |
| Dyspnea | 4 (7.4) |
| Rhinorrhea | 15 (27.8) |
| Sore throat | 20 (37.0) |
| Nausea | 2 (3.7) |
| Diarrhea | 12 (22.2) |
| Abdominal pain | 2 (3.7) |
| Myalgia | 14 (25.9) |
| Headache | 12 (22.2) |
| Anosmia or taste abnormality | 3 (5.6) |
| Laboratory findings | |
| Blood leukocyte count, reference range 4.0–11.0 × $10^9$/L | |
| ≤4.0 × $10^9$/L | 16 (29.6) |
| >4.0 × $10^9$/L | 38 (70.4) |
| Lymphocyte count; reference range 1.0–3.4 × $10^9$/L† | 1.554 ± 0.556 × $10^9$/L |
| Lymphopenia, <1.0 × $10^9$/L | 8 (14.8) |
| Platelet count, reference range 182–369 × $10^9$/L | |
| ≤150 × $10^9$/L | 2 (3.7) |
| >150 × $10^9$/L | 52 (96.3) |
| Hemoglobin; reference range 11.2–15.7 g/dL† | 13.4 ± 1.48 g/dL |
| C-reactive protein level ≥10 mg/L | 5 (9.26) |
| Procalcitonin level ≥0.5 ng/mL | 0 |
| Lactate dehydrogenase ≥250 IU/L | 40 (74.1) |
| Creatinine ≥133 µmol/L | 0 |
| Alanine aminotransferase >40 IU/L | 5 (9.26) |
| Infiltration in chest x-ray | 15 (27.8) |
| Infiltration in computed tomography | 17 (31.5) |
| Bilateral infiltration | 10 (58.8) |
| Unilateral infiltration | 7 (41.2) |
| Predominantly ground glass opacity | 15 (88.2) |
| Predominantly consolidation | 2 (11.8) |
| Supplementary oxygen | 1 (1.9) |

*Characteristics and clinical findings at time of isolation or hospital admission.
†Mean ± standard deviation

**Appendix Table 2.** Attack rate by instructor and sports facility during an outbreak of coronavirus disease associated with fitness dance classes, South Korea, 2020

| Instructor, sports facility | Potentially exposed, no. | Confirmed, no. | Attack rate, % (95% CI) |
|---|---|---|---|
| Instructor A | | | |
| Sports facility 1 | 20 | 14 | 70 (48.1–85.5) |
| Sports facility 2 | 23 | 7 | 30.4 (15.6–50.9) |
| Sports facility 3 | 5 | 1 | 20.0 (3.6–62.5) |
| Sports facility 4 | 10 | 1 | 10.0 (1.8–40.4) |
| Sports facility 5 | 7 | 1 | 14.3 (2.6–51.3) |
| Instructor B | | | |
| Sports facility 6 | 20 | 1 | 5 (0.9–23.6) |
| Sports facility 7 | 9 | 4 | 44.4 (18.9–73.3) |
| Sports facility 8 | 22 | 5 | 22.7 (10.1–43.4) |
| Sports facility 9* | 5 | 3 | 60.0 (23.1–88.2) |
| Sports facility 10 | 18 | 12 | 66.7 (43.8–83.7) |
| Instructor C, sports facility 9 | 25 | 0 | 0 |
| Instructor D | 20 | 4 | 20.0 (8.1–41.6) |
| Instructor E | 26 | 0 | 0 |
| Instructor F | 7 | 4 | 57.1 (25.1–84.2) |
| Total | 217 | 57 | 26.3 (20.9–32.5) |

*Instructor C taught Pilates and yoga in the same facility as Instructor B. Instructor C tested positive for coronavirus disease, but her students did not test positive for coronavirus, possibly because her fitness classes were lower intensity than those of instructors teaching fitness dance classes.

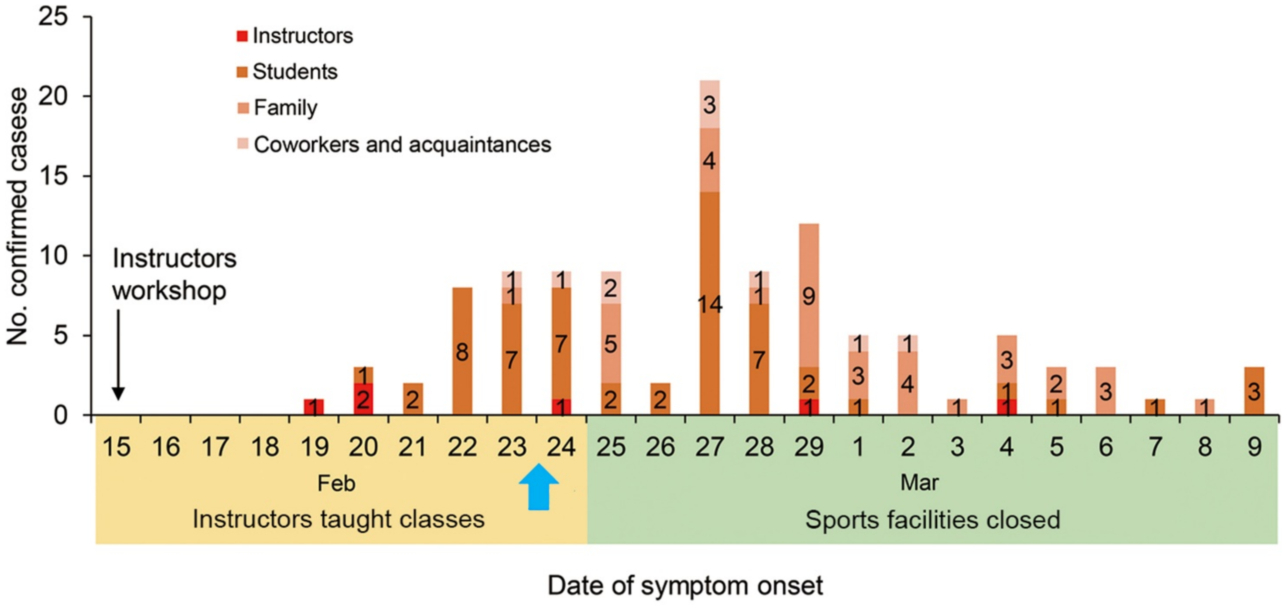


**Appendix Figure 1.** Epidemic curve of laboratory-confirmed coronavirus disease (COVID-19) cases in Cheonan, South Korea, by date of symptom onset and relationship. Blue arrow indicates date that South Korea raised the alert level for COVID-19 to the highest level and requested cooperation for reduction or closure of multi-dense facilities as a part of social and physical distancing policy.

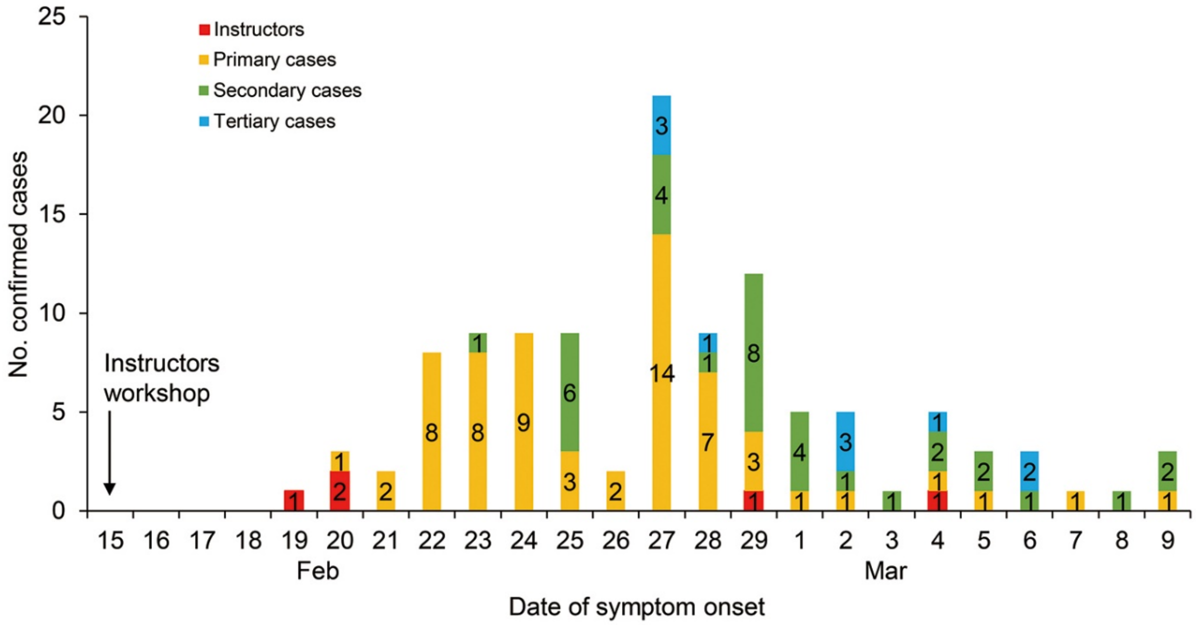


**Appendix Figure 2**. Epidemic curve of laboratory-confirmed coronavirus disease (COVID-19) cases in Cheonan, South Korea, by date of symptom onset and relationship. February 15, 2020 marks the date of a workshop for fitness dance instructors in Cheonan.
